# First reported case of Haemoglobin-M Hyde Park in a Malay family living in Malaysia

**DOI:** 10.17179/excli2016-613

**Published:** 2016-11-07

**Authors:** Tang Yee Loong, Doris Lau Sie Chong, A. Rahman A. Jamal, Nor Azian Abdul Murad, Raja Zahratul Azma Raja Sabudin, Leong Chooi Fun

**Affiliations:** 1 Department of Pathology, UKM Medical Centre, Malaysia; 2 Department of Paediatric, UKM Medical Centre, Malaysia; 3 UKM Medical Molecular Biology Institute, Malaysia

**Keywords:** Hb-M Hyde Park, Hb-M Akita, methaemoglobin, cyanosis, ferric

## Abstract

Haemoglobin (Hb)-M Hyde Park, also known as Hb-M Akita is a rare type of hereditary Hb M due to autosomal dominant mutation of CAC>TAC on codon 92 of β globin gene resulting in the replacement of histidine by tyrosine on β globin chain. This variant Hb has a tendency to form methaemoglobin (metHb). The iron ion in metHb is oxidized to ferric (Fe3+) which is unable to carry oxygen and the patients manifest as cyanosis clinically. A 9-year-old Malay girl was incidentally found to be cyanotic when she presented to a health clinic. Laboratory investigations revealed raised methaemoglobin levels and Hb analysis findings were consistent with Hb-M Hyde Park. β gene sequencing confirmed a point mutation of CAC>TAC on codon 92 in one of the β genes. The family study done on the individuals with cyanosis showed similar findings. A diagnosis of heterozygous Hb-M Hyde Park was made. Patients with this variant Hb usually presented with cyanosis with mild haemolysis and maybe misdiagnosed as congenital heart disease. No further treatment is needed as patients are relatively asymptomatic. Although the disease is harmless in the heterozygous carriers but the offspring of the carriers may suffer severe haemolytic anaemia when the offspring also inherit other β haemoglobinopathies/thalassemia. This can happen due to high prevalence of β thalassemia carrier (3.5-4 %) found in Malaysia. At the time of writing, this is the first case of hereditary Hb-M Hyde Park diagnosed in a Malay family living in Malaysia.

## Introduction

Methaemoglobinemia occurs when blood contain more than 1 % metHb (Saralyn, 2001[8]). Methaemoglobinemia reduces the oxygen-carrying capacity of blood by two mechanisms. First, the ferric iron (Fe3+) in metHb is unable to carry oxygen or carbon dioxide. Second, the conformation of haemoglobin is altered when one or more iron ions have been oxidized and shifts the oxyhaemoglobin dissociation curve to the left (Saralyn, 2001[8]). This shift increases the oxygen affinity of the remaining ferrous (Fe2+) haeme groups to bind oxygen strongly and thus not ready to release oxygen to tissues (Saralyn, 2001[8]). Patients are usually presented with cyanosis and the severity of the symptoms are proportional to the metHb level. We describe a rare case of a Malay family who were presented with cyanosis secondary to Hb-M Hyde Park. 

## Case Report

**Figure 1:**
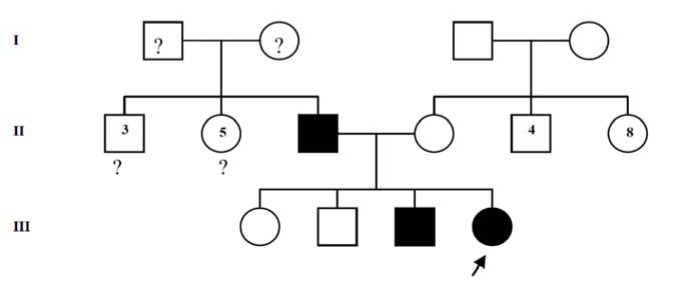
Family tree of patient

A 9-year-old Malay girl was incidentally found to be cyanotic when she presented to a health clinic for acute food poisoning. She was well prior to this and there was no history of reduced effort tolerance, syncopal attack or chest pain. No history of recent drug ingestion was noted. The parents noticed that she had bluish discolouration of the lips and fingernails since the age of 6 years old but did not seek any treatment as she was asymptomatic. Antenatal history was uneventful and she was born term with birth weight of 3 kg. Both her parents were non-consanguineous and she was the youngest out of 4 siblings. Her father and second brother also had cyanosis but were otherwise well while her mother and the other two siblings were normal (Figure 1(Fig. 1)).

Clinically, she was alert and cheerful. She had cyanosis and her SpO2 on room air was 60-65 %. Otherwise, she was not tachycardic or tachypnoeic and her blood pressure was normal. She was thriving with her growth parameters at the 50th percentile on the growth chart. Examination of the cardiovascular system revealed normal first and second heart sounds with no murmur heard. Echocardiogram done revealed normal intracardiac anatomy and function. Other systems were unremarkable. 

**Figure 2:**
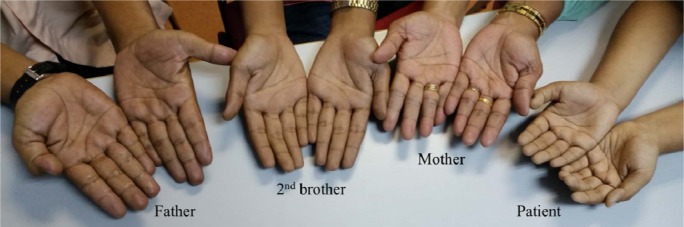
(From right) Skin colour of patient, her mother, second brother and father

**Figure 3:**
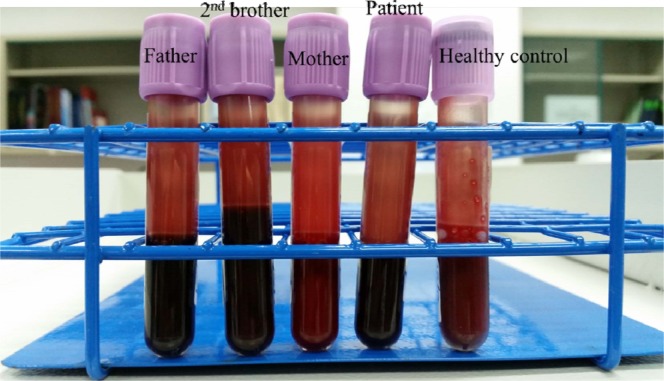
(From right) Venous blood in ethylenediaminetetraacetic acid (EDTA) tube of healthy control (normal colour), patient (dark brown colour), patient's mother (normal colour), patient's second brother and patient's father (dark brown colour)

**Figure 4:**
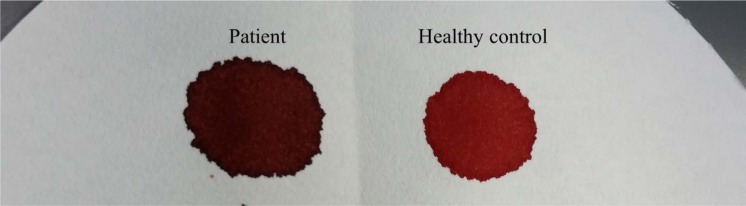
(From right) Venous blood's colour of healthy control (bright red) and patient (dark brown) with exposure of atmospheric oxygen for 10 minutes

Family study was performed on the individuals with cyanosis (Figure 2(Fig. 2)). The venous blood samples were collected and showed distinct dark brown colour from the affected family members as opposed to normal colour from the healthy control and unaffected mother (Figure 3(Fig. 3)). A drop of patient's venous blood was placed onto filter paper with the exposure of atmospheric oxygen for 10 minutes. The blood's colour remained as dark brown because the ferric iron (Fe3+) in metHb was unable to carry oxygen despite oxygenation. In contrast, a drop of healthy control's venous blood turned to bright red after exposure of atmospheric oxygen (Figure 4(Fig. 4)) due to conversion of deoxygenated Hb to oxygenated Hb.

**Figure 5:**
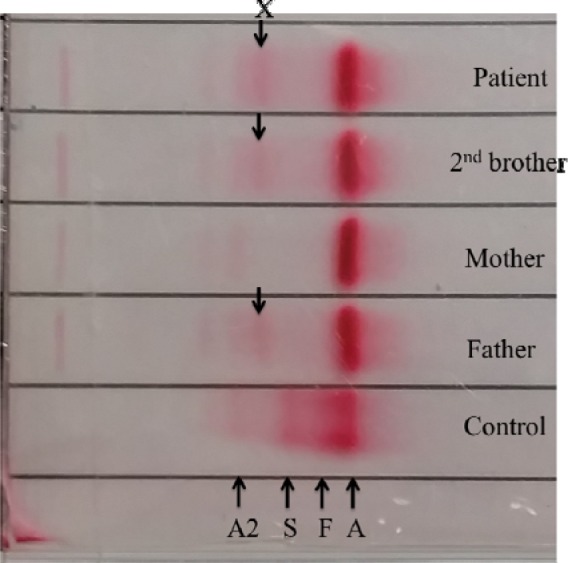
Abnormal band (X) was seen in between HbA2 and HbS at alkaline pH.

**Figure 6:**
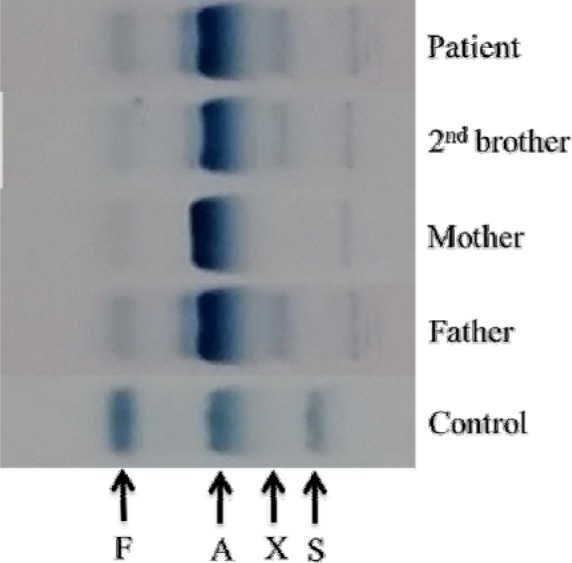
Abnormal band (X) was seen in between HbA and HbS at acidic pH.

**Figure 7:**
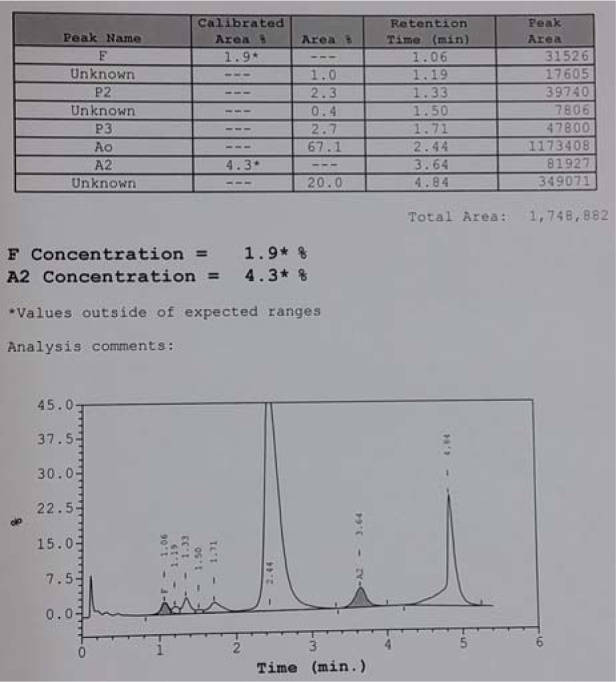
HPLC of patient's haemoglobin showed an unknown peak at retention time of 4.84 min.

**Figure 8:**
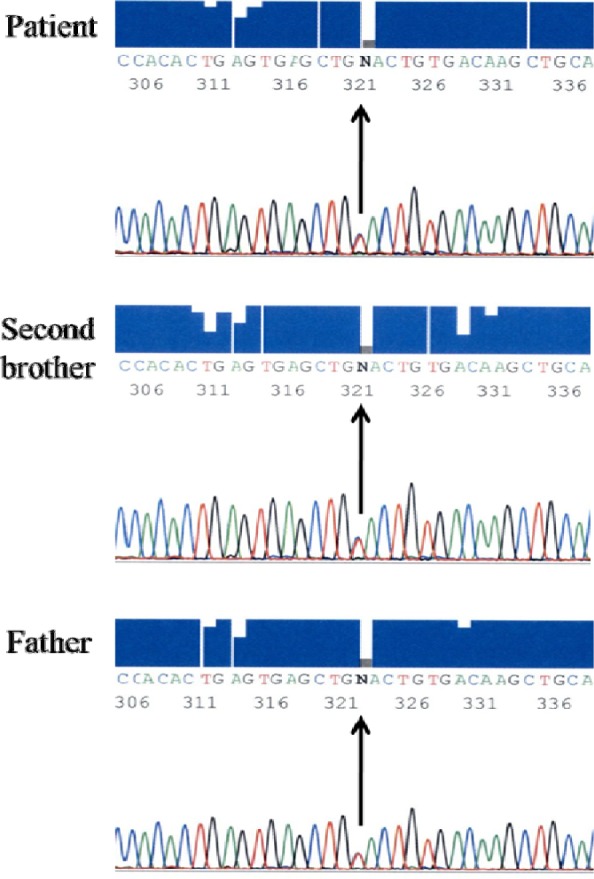
β gene sequencing revealed a heterozygous point mutation of CAC>TAC on codon 92 at β globin genes.

The patient's full blood picture was normal but showed microcytic hypochromic erythrocytes in her father and brother. Patient and her father had slightly elevated of serum total bilirubin (30 µmlo/L and 38 µmol/L respectively) but the brother's result was normal (20 µmol/L). Blood gas analysis displayed raised metHb levels (Patient: 13.7 %, Father: 11.3 %, Brother: 11.9 %). Hb electrophoresis at alkaline pH showed abnormal band in between HbA_2_ and HbS (Figure 5(Fig. 5)) while at acidic pH showed abnormal band in between HbA and HbS (Figure 6(Fig. 6)). Capillary electrophoresis (CE) of Hb showed variant Hb (Patient: 5.3 %, Father: 4.1 %, Brother: 3.7 %) at zone 4. High-performance liquid chromatography (HPLC) of Hb demonstrated an unknown peak at retention time of 4.84 min with the levels of 20 % in patient (Figure 7(Fig. 7)) and 16.7 % in brother but no result for father due to insufficient sample. Besides, there was slightly raised of HbA_2_ in three of them (Patient: HPLC 4.3 %, CE 4.7 %; Father: CE 4.2 %; Brother: HPLC 4.1 %, CE 4.4 %). The HbF level was also slightly elevated in them (Patient: HPLC 1.9 %, CE 1.5 %; Father: CE 1.3 %; Brother: HPLC 1.9 %, CE 1.8 %). Deoxyribonucleic acid (DNA) analysis of deletional α thalassemia by multiplex polymerase chain reaction (PCR) showed αα/αα in patient but αα/-α3.7 in her father and brother. β gene sequencing confirmed a point mutation of CAC>TAC on codon 92 in one of the β genes in three of them (Figure 8(Fig. 8)). The final diagnosis in the patient was heterozygous Hb-M Hyde Park. Her father and brother were diagnosed as heterozygous Hb-M Hyde Park with concomitant heterozygous α+ thalassemia trait. Genetic counselling was given to the affected family members. No specific treatment is required although cyanosis was present, the patients were relatively asymptomatic.

## Discussion

The most common causes of metHb are acquired rather than congenital. Drugs that cause metHb include anti-infective such as sulfonamides, dapsone, chloroquine, primaquine, local anesthetics such as benzocaine, prilocaine, lidocaine and others. Congenital causes include hereditary Hb M and deficiency in the nicotinamide adenine dinucleotide (NADH) metHb reductase enzyme system (Saralyn, 2001[8]). Hb M variants have a tendency to form metHb, produced by the oxidation of ferrous (Fe2+) in haem to ferric (Fe3+).

Hb-M Hyde Park was first discovered in USA in 1966 in a black patient (Heller et al., 1966[4]). At the same year, a patient with congenital cyanosis living in Japan was found to have Hb-M Akita (Shibata et al., 1976[10]). Amino acid analysis of abnormal peptides showed that both Hb-M Hyde Park and Hb-M Akita were the same disease with histidine substituted by tyrosine at 92 residues of the β chain (Shibata et al., 1968[9]). Subsequently, several rare cases were reported world widely. Hb-M Hyde Park was reported in a 26-year-old Indian man recently (Upadhye et al., 2015[12]). The patients of Hb-M Hyde Park were detected in some parts of Japan by clinical examination rather than by Hb survey (Harano, 1999[3]). Case of Hb-M Hyde Park was also reported in Soviet Union before the state dissolved into current Russia and other countries (Kazanets et al., 1988[5]). So far all the reported cases were in hereditary pattern except two cases. The first case of de novo mutation was reported in a 10-year-old Caucasian girl of Norwegian-German ancestry who was presented with cyanosis since four-month-old but none of the parents had cyanosis (Stamatoyannopoulos et al., 1976[11]). The second case of de novo mutation was reported in a girl who was found to have 16 % metHb but the parents and sister showed no abnormality (Rotoli et al., 1992[7]). All the reported cases were in heterozygous form and until now there is no reported case of homozygous Hb-M Hyde Park. The homozygous form of Hb-M Hyde Park is believed to be lethal and incompatible with life due to presence of large quantity of metHb. The carriers of Hb-M Hyde Park usually presented with cyanosis and mild haemolysis (Upadhye et al., 2015[12]; Shibata et al., 1976[10]). Sometimes they can be misdiagnosed as congenital heart disease (Stamatoyannopoulos et al., 1976[11]). No specific treatment is required as patients are relatively asymptomatic.

The patients in our case report demonstrated the typical history of cyanosis and investigations revealed presence of metHb level (11.3 %-13.7 %), distinct dark brown blood despites oxygenation, the results of Hb analysis and β gene sequencing were consistent with Hb-M Hyde Park. We noticed the patients have mildly raised HbA_2_ and HbF which was also observed with mildly raised HbA_2 _(5 %) but not HbF (0.9 %) by HPLC method in the previous report (Upadhye et al., 2015[12]). Mildly raised HbA_2_ is usually observed in patients with β thalassemia trait or HbE trait (Hafiza et al. 2012[2]) while mildly raised HbF is seen in patients with β thalassemia trait (Gibney et al., 2008[1]). Hb A_2_ or HbF is raised as a compensation that increases the synthesis of δ or γ globin chain when there is a reduction of β chain synthesis (Reisner and Reisner, 2016[6]; Gibney et al., 2008[1]). However, the exact reason of mildly raised HbA_2 _and HbF level in our patients with Hb-M Hyde Park is not known and further study is necessitated. Perhaps this variant Hb carries minor effect of thalassemia that decrease synthesis of β^M-Hyde Park ^globin chain. 

## Conclusion

In conclusion, the diagnosis is challenging due to rarity of the disease. Clues to the diagnosis include the patient who often present as cyanosis since childhood, relatively asymptomatic, family history of cyanosis and cyanosis does not ameliorate with the administration of oxygen. Although the disease is harmless in the heterozygous form but inheritance of other β haemoglobinopathies / thalassemia gene can give rise to significant morbidity. The prevalence of β thalassemia carrier is high (3.5-4 %) in Malaysia. Child produced by a parent with Hb-M Hyde Park and another parent with β thalassemia trait may suffer severe haemolytic anaemia. Therefore, proper genetic counselling must be given to the affected family members. At the time of writing, this is the first case of hereditary Hb-M Hyde Park diagnosed in a Malay family living in Malaysia.
